# The effects of a long-term care walking program on balance, falls and well-being

**DOI:** 10.1186/1471-2318-12-76

**Published:** 2012-12-18

**Authors:** Vanina PM Dal Bello-Haas, Lilian U Thorpe, Lisa M Lix, Rhonda Scudds, Thomas Hadjistavropoulos

**Affiliations:** 1 School of Rehabilitation Sciences, McMaster University, 1400 Main Street West, 403/E, Hamilton, Ontario, L8S 1C7, Canada; 2 Department of Community Health & Epidemiology, College of Medicine, 103 Hospital Drive, Royal University Hospital, Saskatoon, SK, S7N 0W8, Canada; 3 Department of Psychiatry, College of Medicine, 103 Hospital Drive, Saskatoon City Hospital, 701 Queen Street, Saskatoon, Saskatchewan, S7K 0M7, Canada; 4 Department of Community Health Sciences, Faculty of Medicine, University of Manitoba, Winnipeg, Manitoba, R3E 0W3, Canada; 5 School of Physical Therapy, University of Saskatchewan, 1124 College Drive, Saskatoon, Saskatchewan, S7N 0W3, Canada; 6 Department of Psychology, University of Regina, Regina, Saskatchewan, S4S 0A2, Canada

**Keywords:** Walking, Long-term care, Longitudinal studies

## Abstract

**Background:**

The effects of a regular and graduated walking program as a stand-alone intervention for individuals in long-term care are unclear. Exercise and fall prevention programs typically studied in long-term care settings tend to involve more than one exercise mode, such as a combination of balance, aerobic, strengthening, and flexibility exercises; and, measures do not always include mental health symptoms and behaviors, although these may be of even greater significance than physical outcomes.

**Methods/design:**

We are randomly assigning residents of long-term care facilities into one of three intervention groups: (1) *Usual Care Group* - individuals receive care as usual within their long-term care unit; (2) *Interpersonal Interaction Group* - individuals receive a comparable amount of one-on-one stationary interpersonal interaction time with study personnel administering the walking program; and, (3) *Walking Program Group* – individuals participate in a supervised, progressive walking program five days per week, for up to half an hour per day. Assessments completed at baseline, 2 and 4 months during intervention, and 2 and 4 months post-intervention include: gait parameters using the GAITRite® computerized system, grip strength, the Berg Balance Scale, the Senior Fitness Test, the Older Adult Resource Services Physical Activities of Daily Living, the Geriatric Depression Scale Short Form, the Cornell Scale for Depression in Dementia, the Revised Memory and Behavior Problems Checklist, the Short Portable Mental Status Questionnaire, the Coloured Analogue Scale, pain assessment scales, and the number and nature of falls. Sophisticated data analytic procedures taking into account both the longitudinal nature of the data and the potential for missing data points due to attrition, will be employed.

**Discussion:**

Residents in long-term care have a very high number of comorbidities including physical, mental health, and cognitive. The presence of dementia in particular makes standardized research protocols difficult to follow, and staff shortages, along with inconsistencies related to shift changes may impact on the accuracy of caregiver-rated assessment scales. Practical challenges to data collection validity and maintenance of inter-rater reliability due to the large number of research staff required to implement the interventions at multiple sites are also posed.

**Trial registration:**

ClinicalTrials.gov NCT01277809

## Background

Routine physical activity, as an essential component of healthy and successful aging, is a means of maintaining mobility and functional abilities in aging adults [1-3]. A common challenge for long-term care (LTC) residents is decreased mobility, the ability to move independently within one’s environment [4], or immobility, with an estimated 89% of nursing home residents experiencing some level of mobility limitation [5]. Decreased mobility and immobility are associated with a variety of physical and psychosocial impairments, including depression, anxiety, feelings of isolation, activity limitations, and complications in almost every body organ system [6-10]. In addition, decreased mobility and immobility lead to a devastating spiral of further disability and health problems. While it is thought that decreased mobility or immobility are natural consequences of aging, researchers have found that frail older adults have a much greater potential for increased mobility, and physical, psychological and functional improvements than previously thought possible [11-14].

Clinical trials of exercise interventions, including strength, endurance, balance and functional training, or combination training, in LTC settings have led to significant positive effects on health outcomes [11,12,15-19]. Interventions that are both efficient and effective in maximizing resident’s mobility and functional abilities are essential, particularly in chronic care settings. However, sustainable approaches to incorporating physical activity or mobility interventions that are acceptable to both residents and staff, and that can be inclusive of a large proportion of those living in LTC facilities have not yet been identified [20]. Walking, as an intervention, is ideal in its simplicity as a means of improving mobility, strength, and endurance, and in its ability to be integrated into daily care routines. Although limited by non-random samples and unblinded outcome measurement, earlier studies of walking programs have been found to be beneficial in improving walking endurance [21], ambulation status [22], and urinary incontinence [23]. Combined walking and talking interventions have also been found to be beneficial. Improved communication performance in LTC residents who participated in a walking and talking program compared to those who engaged in talking alone has been reported [24]. Tappen, Roach, Applegate and Stowell (2000) compared combined walking (30 minutes of self-paced assisted walking three times per weeks for 16 weeks) and talking (30 minutes of conversation) to walking alone and talking alone. Pre-test and post-test measurement found less decline in the distance walked in six minutes in the combined treatment group – 2.5% decline versus 18.8% decline (talking group) and 20.7% decline (walking group) [25]. Building on prior work, Cott and colleagues (2002) compared walking and talking in pairs (30 minutes 5 times per week for 16 weeks) to talking alone (conversation in pairs) and no-intervention. The walking and talking intervention did not produce statistically significant differences in communication, ambulation or functional status. The authors noted that for those with moderate to severe dementia, the dual task of walking and talking with another resident (“pair walking, talking”) may be too much stimulation, leading to multiple stops and starts of the walk and may have resulted in insufficient walking [26]. In summary, the extent of the research-to-date examining the effects of walking programs is equivocal and limited. Previous studies have focused on selective outcome measures and have not examined the effects of a walking program on falls, pain, psychosocial function and behavior.

In this paper, we describe a study protocol assessing the effectiveness of an individualized, progressive, walking program (which inevitably involves human interaction) compared to usual care and to a human interaction condition (no walking) in individuals residing in long-term care facilities. Our study protocol addresses methodological limitations of previous LTC walking studies; specifically, we: 1) have included a broader array of physical, physiological and psychosocial outcome measures, including measures of strength, balance, flexibility, blood pressure, heart rate, weight, height (total and knee-floor), waist, upper arm and calf circumference, pain, activities of daily living (ADL), behaviour, falls, hospitalizations, depression, cognition, psychotropic medication use; 2) will examine gait speed and gait characteristics using a computerized system; and, 3) are ensuring that participants are receiving adequate Vitamin D, 1000 IU per day, based on most recent recommendations regarding the use of Vitamin D in older persons residing in LTC settings [27].

## Methods/Design

### Design

This study is a prospective, randomized, three-group design with multiple dependent variables. The three groups include: (1) Participants receiving care as usual (*Usual Care Group*); (2) Participants receiving stationary 1:1 interaction time with research personnel in order to control for the interpersonal interaction (*Interpersonal Interaction Group*); and, (3) Participants engaging in an individualized, progressive, one-to-one supervised walking program five days per week (*Walking Program Group*).

### Ethics

The study has been approved by all necessary research ethics boards (i.e., university, as well as health region).

### Target population, inclusion and exclusion criteria

Participants over the age of 60 are being recruited from long-term care facilities. To be included in the study, residents must be able to follow simple instructions, must be able to ambulate with or without a walking aid for at least 10 metres, must be available Monday-Friday for interventions and be willing to participate in a 5-day per week walking program over a 4-month period. Residents who have experienced a recent cardiovascular event (within past 6 months) or have severe, mobility limiting arthritis, cardiac instability, a severe, mobility limiting vestibular disorder, uncontrolled hypertension, uncontrolled epilepsy, a recent fracture (past 4 months), who are unable to satisfactorily comply with the protocol requirements, who have had a recent admission into an acute care facility (past 4 months), who are scheduled for surgery or hospitalization in the next 6 months or who are participating in another regular exercise program (half an hour or more, three or more times per week ) aimed at improving balance or strength will be excluded from the study.

### Sample size

Sample size calculations assumed a moderate standardized effect size of 0.40 [28], a small value for the intra-class correlation (i.e., the proportion of variation accounted for by LTC units) of 0.05, and α = .05, power = .80. Sample size calculations determined that a sample size of 129 total participants will provide ample power for repeated measures analyses [29]. A balanced design will be adopted, with equal numbers of participants in each group. We will collect data from an additional 50 participants in order to account for attrition due to refusal or inability to continue with the protocol. As well, morbidity and mortality may account for some attrition.

### Recruitment

The investigators will meet with each of the LTC facilities’ administrators and staff to inform them of the study, inclusion and exclusion criteria, and study procedures and interventions. Staff is asked to identify potential/eligible participants who are then approached by research assistants and asked if they wish to participate in the study. If the potential participant agrees to participate, this is considered to be assent, regardless of capacity for full, informed consent. The research assistant then explains the study, asking the participant questions to assess full understanding of the protocol including potential benefits and adverse effects. If the participant clearly understands the protocol, he/she will is invited to sign a full consent form. If the participant does not have full understanding of the protocol, or the capacity of the subject is unclear, the research assistant asks permission to contact the substitute decision-maker (SDM) who normally consents to health care decisions. If the participant declines, he/she will not be entered into the study. If the participant agrees, the SDM is approached and the protocol is explained in detail. The consent form is signed by only the SDM when the participant clearly has no capacity, and by both the participant and the substitute decision-maker when capacity is unclear or diminished

### Procedures

After completing informed consent to participate in the study (or proxy consent by the appropriate SDM), all participants regardless of group will undergo a baseline assessment (see outcome measures section) conducted by trained study personnel prior to group allocation. Prior to enrolment in the specific study arm, family physicians are asked to optimize vitamin D levels, which will generally require obtaining vitamin D levels and prescribing 1,000 IU Vitamin D (if the participant is not already taking 1,000 IU or more) to control for potential influences on our study outcomes, and to be consistent with current recommendations [27]. Adherence with Vitamin D recommendations is monitored and reasons for intake problems are recorded.

Physical measures including respiratory rate, lying (after three to five minutes) and standing (after one minute) blood pressure, heart rate, as well as full height, knee-floor height, waist and calf circumference and weight are also collected at the beginning of the study, and except for height, measured also at week 8, week 16, week 24 and week 32, and whenever a medical concern arises. All adverse events (including falls, hospitalizations and surgeries) charted in nurses progress notes are collected, as are most recent full Resident Assessment Instrument-Minimum Data Set (RAI-MDS) and RAI-MDS updates, and the full, monthly medication administration record.

A licensed physiotherapist will assess the participant for fall risk and assign a visual analogue risk score ranging from negligible fall risk to highest fall risk, commenting on the reasons for the risk score assigned.

### Intervention

Participants within each facility are randomly assigned to one of three intervention groups. The intervention period is four months. Participants in the control group receive the usual care of their LTC unit throughout the four month time period, with the exception of scheduled outcome assessments. Participants in the Interpersonal Interaction group receive an approximately equivalent interpersonal interaction time (up to 30 minutes per day, five days per week) with research personnel as those in the Walking Program group. The interaction time occurs with the participant stationary. The participants in the Walking Program group receive an individualized, progressive, one-to-one supervised walking program provided by study personnel and supervised by a licensed physiotherapist. Each participant in this group walks once per day (up to half an hour), five days per week. The initial distance walked is initially determined for each individual based on how far the person can ambulate before visibly becoming fatigued or short of breath, reporting pain or requesting to sit down or rest. The distance walked and the intensity of the walking program during the four month period is gradually increased, as tolerated by the individual.

### Outcome measures

Outcome measures are completed at baseline. Re-assessment occurs after two months and four months of the intervention period, and at two and four months after the intervention period. The assessment of the outcome measures will be conducted by study personnel who are blind with respect to group allocation. The outcome measures include:

(1) Seniors Fitness Test (SFT) [30]. The SFT is a six-item battery, with demonstrated intra-rater and test-retest reliability and validity in older adults [30]. The SFT includes measures of dynamic upper (arm curls) and lower extremity strength (30-second sit-to-stand) and flexibility, aerobic endurance (2-minute step test), and mobility (8-ft walk test) [30].

(2) Berg Balance Scale (BBS). The BBS, a 14-item battery that assess a person's ability to safely perform several common daily living tasks, and is widely used as a measure of balance performance and fall risk assessment [31-33]. Performance on each item is rated on a scale from 0 (cannot perform the task) to 4 (normal performance of the task) and the maximum obtainable score is 56. The BBS has been reported to have excellent interrater and intrarater reliability and concurrent and discriminant validity [34].

(3) Grip strength. Grip strength is a reliable measurement when standardised methods and calibrated equipment are used, independent of evaluator or type of dynamometer, [35] and has been found to be a powerful predictor of disability, morbidity and mortality [36-38]. We will measure grip strength using a hand-held dynamometer using standard testing procedures. Three maximal repetitions (with 30 second rest between attempts) will be recorded for the dominant and non-dominant hand.

(4) Older American Resource Services (OARS) Activities of Daily Living Scale. The OARS is a brief, valid, and reliable instrument that is used as a clinical assessment of functional status [39]. The OARS ADL scale includes seven basic ADL items (eating, dressing and undressing, grooming, walking, getting in and out of bed, bathing, and continence), and seven instrumental ADL items (using the telephone, travel, shopping, meal preparation, housework, taking medicine, and management of finances) [39]. We will record the scores for the BADL items.

(5) Cornell Scale for Depression in Dementia (CSDD). We are assessing depression using the CSDD, a well-validated scale, with high sensitivity [40,41], and high inter-rater reliability and internal consistency [41]. The CSDD is designed for the assessment of depression in individuals with dementia and examines five areas: Mood-Related Signs, Behavioral Disturbances, Physical Signs, Cyclic Functions, and Ideational Disturbances [41].

(6) Geriatric Depression Scale (GDS) Short Form. We are also examining depression using the GDS Short Form, a well-known screen for depression in older adults which is widely used in community and LTC settings [42,43].

(7) Revised Memory and Behavior Problems Checklist (RMBPC). Behavioral problems will be assessed using the RMBPC, a valid and reliable 24-item measure for people with dementia, that includes three subscales, Memory-Related Problems, Affective Distress, and Disruptive Behaviour [44].

(8) Short Portable Mental Status Questionnaire [45]. The Short Portable Mental Status Questionnaire (SPMSQ) is a 10-item examination that has been found reliable and valid in distinguishing demented subjects from cognitively intact subjects. It is quick to administer and does not require much training, and we are using the SPMSQ to assess mental status.

(9) Coloured Analogue Scale [46]. This scale is being used to assess pain via patient self-report. Specifically, pain is rated by moving a plastic glide along a 14.5 cm long grid, varying in colour and width. That is, the scale is 1 cm wide at the bottom gradually widening to 3 cm at the top. Its colour is light pink at the bottom and progresses to a deep red colour at the top. The ends of the scale are anchored by the polar opposites “no pain” and “most pain”. As such, participants are presented with several visible cues for scaling the severity of their pain: length, width, colour and the anchoring words. The back of the scale has numbers ranging from 0 to 10 that are used by the assessor for the recording of pain intensity. This measure has been shown to be a valid index of self-reported pain with seniors who have mild to moderate dementia in that scores increase from baseline to painful situations [47].

(9) Gait speed (6 metre walk test). Participants will be asked to walk at their usual walking speed over a distance of 6 metres on the sensor mat of the GAITRite® system. The GAITRite® system (CIR Systems Inc., Clifton, NJ) is a portable walkway device, found valid and reliable for measuring selected spatial and temporal gait parameters [48-52] and allows concurrent videotaping of ambulation through a video camera interface. An additional two metres of walking will be added on each side to accommodate for acceleration and deceleration. The average of two attempts will be used to calculate walking speed in feet/second. Gait parameters, including gait speed, will be recorded.

### Other data collection

In order to control for confounding variables and to characterize the sample, the admission and most recent quarterly Resident Assessment Instrument-Minimum Data Set (RAI-MDS) [53] assessment data will be extracted. The RAI-MDS is a standardized LTC assessment tool designed to improve the care given to long-term care residents and it is used widely in Canada. The RAI-MDS data will be linked to the participants’ study ID number. The following RAI-MDS data will be included in the analysis: date of birth, race/ethnicity, gender, highest level of education, diagnoses on admittance, medications, health conditions; Cognitive Performance Scale; Depression Rating Scale; Index of Social Engagement; ADL Self-Performance Hierarchy Scale; Pain; Continence; Oral/Nutritional status; Communication; Mood and Behaviour Patterns; Physical Functioning and Structural problems; Activity Pursuit Patterns; Special Treatments and Procedures.

The incidence and nature of falls will be prospectively being collected using data from the Saskatoon Health Region Falls documentation protocol for the period of the intervention (four months) and for four months following intervention. Clinical staff records the nature and location of the fall, activity and symptoms at the time of the fall, and injuries resulting from the fall. Research assistants make copies of all falls records and add this to the research chart.

### Study organization

The clinical director of the study (principal investigator) is responsible for research personnel hiring and training, informed consent procedures, participant selection, clinical assessments, management of adverse events, data management, and general oversight of study procedures. A clinical research coordinator is carrying out many of the day to day tasks under the direction of the clinical director. A co-investigator will be responsible for the data cleaning and analyses.

### Data analysis

We hypothesize that participants taking part in the walking program will demonstrate maximal benefits compared to the no treatment control group (usual care) and the participants receiving only social interaction. We hypothesize that benefits of the walking program will include decreased fall rates, and improved balance, endurance, strength, mood, behaviour, activities of daily living and quality of life indices. As research has found that pleasant activities improve resident mood [54], we also hypothesize that participants in the social interaction group will demonstrate improvements in mood and other indices of quality of life.

The data will be analyzed using a random-effects multiple regression model [55]. The between-subjects factor will be condition (i.e., treatment and two control conditions). A random intercept for time (i.e., 5 measurement occasions) will be included to account for within-subject variation due to the repeated measurements. We will also evaluate model fit when a random slope for time is included in the model. Model fit will also be evaluated when the time x group interaction is included in the model. Additional covariates that may be included in the model are clinical measures from the RAI-MDS, such as the presence of comorbid conditions. Evaluation of model fit will be conducted using likelihood ratio tests and the Aikake Information Criterion [55]. Proportions of participants in the number of fall categories (e.g., non-faller, one-time faller, multiple faller) will be assessed using Pearson’s chi-square statistics.

## Discussion

The nature of the study overall, the study population, and the study design necessitates co-operation from and collaboration among the LTC administrative and health care staff, family members, family physicians and research personnel. Early involvement of key stakeholders, frequent communication and updates, as well as timely responsiveness to issues and concerns are critical to the success of this study. Implementing a walking intervention study in a population with varying degrees of cognitive and physical impairments who reside in LTC is not without its challenges. The large majority of the individuals to be recruited will present with some level of cognitive impairment. Numerous studies have led to the conclusion that people with moderate cognitive impairments (defined as Mini Mental Status Examination [56] scores of 12/13) are able to respond consistently to questions about choices and their involvement in decisions about daily living [57-60]. However, some residents will have cognitive impairments that are so severe that they cannot provide informed consent to participate in the study. An additional step in the recruitment protocol then has been to obtain proxy consent from a designated substitute decision maker or in the cases where capacity to consent is not clear, obtain both resident and proxy consent. Meeting with the LTC staff to discuss the study is an important first step, as the staff being asked to identify potential/eligible participants and assess residents to determine if potential participants are capable of providing informed consent. Obtaining consent and consent processes can be lengthy and difficult, even with individuals without cognitive impairments, and with our study population we have an additional layer of complexity to the study procedures. Not only do cognitive impairments affect the consent processes, but difficulty with data collection, in particular with those with severe impairments, is expected.

We initially structured the walking intervention to be comprised of two 30-minute walks per day, but quickly realized that the feasibility of this approach would not work in the participating LTC facilities. Long-term care facilities, by nature, have limited organizational flexibility with structured times for meals, activities, medication administration, vitals checks and so on, which limits the amount of time to engage the participants in the intervention. These conflicting daily activities provide a very narrow window of opportunity for many aspects of the study, including obtaining consent, baseline assessments and re-assessments and intervention implementation. Additional LTC contextual and environmental factors, including staff-to-resident ratios, caseload mix, organization of services, research readiness, staffing shortages, and viral outbreaks have all impacted on the study procedures and day-to-day implementation.

A considerable amount of time spent on Research Assistant (RA) training and standardization to ensure the walking intervention is safe, effective and similar among the RAs who are hired for the walking intervention.

Interaction and communication with individuals with cognitive impairments are important considerations for conducting study assessments and carrying out the interventions. Creating a personal connection with the individual; using appropriate facial expressions, eye contact, and tone of voice; using one-step commands; and, incorporating appropriate and consistent cueing in a progressive manner (e.g., verbal and visual cueing), demonstration, tactile guidance, and if necessary, physical assistance are useful strategies that have been reported in the literature and that need to be incorporated into the assessment and intervention protocols. Evaluators and research staff need to be trained in these strategies in order to maximize the success of interactions, and to ensure consistency of testing procedures and intervention implementation. Because the research sites include several LTC facilities, RA scheduling is a challenge. Intermittent quarantining of facilities due to outbreaks further challenges research staff to supply consistent interventions. Last, because of the length of the study, RA turnover because of competing academic and career priorities necessitates much time to be spent on quality control.

## Conclusion

Despite the challenges, through this study, insights may be garnered regarding benefits of a walking program and practical intervention implication insights. Determining if the intervention is beneficial may facilitate improved adherence with walking programs in LTC facilities. Improved resident quality of life, psychosocial function, functional abilities and fewer falls all have the potential to lead to favourable financial and health care system outcomes over the longer term.

## Abbreviations

ADL: Activities of daily living; BBS: Berg Balance Scale; CSDD: Cornell Scale for Depression in Dementia; GDS: Geriatric Depression Scale; LTC: Long-term care; OARS: Older American Resource Services; RA: Research assistant; RAI-MDS: Resident Assessment Instrument-Minimum Data Set; RMBPC: Revised Memory and Behaviour Problems Checklist; SDM: Substitute decision-maker; SFT: Seniors Fitness Test; SPMSQ: Short Portable Mental Status Questionnaire.

## Competing interests

Financial competing interests

· In the past five years have you received reimbursements, fees, funding, or salary from an organization that may in any way gain or lose financially from the publication of this manuscript, either now or in the future? Is such an organization financing this manuscript (including the article-processing charge)? If so, please specify.

⊠ No □ Yes

· Do you hold any stocks or shares in an organization that may in any way gain or lose financially from the publication of this manuscript, either now or in the future? If so, please specify.

⊠ No □ Yes

· Do you hold or are you currently applying for any patents relating to the content of the manuscript? Have you received reimbursements, fees, funding, or salary from an organization that holds or has applied for patents relating to the content of the manuscript? If so, please specify.

⊠ No □ Yes

· Do you have any other financial competing interests? If so, please specify.

⊠ No □ Yes

Non-financial competing interests

· Are there any non-financial competing interests (political, personal, religious, ideological, academic, intellectual, commercial or any other) to declare in relation to this manuscript? If so, please specify.

⊠ No □ Yes

## Authors’ contributions

VDB-H is corresponding author. She substantially contributed to the conception and design of the study and data collection instruments and research procedures; drafted and revised the manuscript. LUT substantially contributed to the conception and design of this study and data collection instruments and research procedures; hired, trained and supervised the research coordinator and research assistants, maintained clinical responsibility for all phases of the study; critically reviewed drafts of the manuscript for important intellectual content. LML substantially contributed to the data analyses and research procedures; critically reviewed drafts of the manuscript for important intellectual content. RS substantially contributed to the conception and design of this study and data collection instruments and research procedures; critically reviewed drafts of the manuscript for important intellectual content. TH substantially contributed to the conception and design of this study; critically reviewed drafts of the manuscript for important intellectual content. All authors read and approved the final manuscript.

## Pre-publication history

The pre-publication history for this paper can be accessed here:

http://www.biomedcentral.com/1471-2318/12/76/prepub
